# Mediterranean Diet and Workplace Health Promotion

**DOI:** 10.1007/s12170-014-0416-3

**Published:** 2014-10-10

**Authors:** Maria Korre, Michael A. Tsoukas, Elpida Frantzeskou, Justin Yang, Stefanos N. Kales

**Affiliations:** 1 Environmental & Occupational Medicine & Epidemiology, Harvard School of Public Health, Boston, MA USA; 2 Occupational Medicine, The Cambridge Health Alliance, Harvard Medical School, Cambridge, MA USA; 3 Division of Endocrinology and Metabolism, McGill University Health Center, Montreal, Quebec Canada; 4 Occupational Medicine Department, Evangelismos Hospital, Medical School, University of Athens, Athens, Greece; 5 Department of Internal Medicine, St Elizabeth’s Medical Center, Tufts Medical School, Brighton, MA USA

**Keywords:** Mediterranean diet, Workplace, Dietary habits, Occupational health, Health promotion, Coronary heart disease, Myocardial infarction, Cardiovascular disease, Diabetes, Metabolic syndrome, Dyslipidemia, Cancer, Mortality

## Abstract

Analytical and experimental studies confirm relationships between the consumption of certain foods and cardiovascular disease, diabetes, and cancer. Mediterranean diet patterns have long been associated with a reduced risk of major diseases and many favorable health outcomes. Data from observational, longitudinal, and randomized controlled trials have demonstrated that Mediterranean-style diets can improve body mass index and body weight, reduce the incidence of diabetes mellitus and metabolic syndrome risk factors, decrease cardiovascular morbidity and coronary heart disease mortality, as well as decrease all-cause mortality. Recently, efforts have attempted to improve dietary habits in the workplace, by modifying food selection, eating patterns, meal frequency, and the sourcing of meals taken during work. Evidence supporting the Mediterranean diet and the potential cardioprotective role of healthier diets in the workplace are reviewed here, and promising strategies to improve metabolic and cardiovascular health outcomes are also provided.

## Introduction

The relationship between nutrition and health has been known since ancient times. Over the last 50 years, the impact of specific dietary patterns and foods of higher nutritional quality on the health and well-being of populations has been demonstrated with ecologic, observational, case–control studies, prospective cohorts, and randomized clinical trials. This considerable body of evidence has established that diet and dietary factors play strong roles in the prevention/development of chronic diseases, such as diabetes, cardiovascular disease, cancer and dementia, as well as mortality [1–3]. Currently, major medical associations such as the World Health Organization place an ever-increasing emphasis on the role of diet in preventing non-communicable diseases [4]. The traditional Mediterranean diet (MD) has been consumed in the Mediterranean basin for thousands of years, but only formally described by Ancel Keys in the 1960s [5]. It includes a high intake of virgin olive oil, fruits, vegetables, other plant proteins and fibers (nuts and legumes), unrefined whole grains, and fish; a moderate intake of dairy, eggs, and lean meats; moderate alcohol intake with meals (usually as wine); and low red meat, refined carbohydrate, and sweet consumption [6–8].

Data both from analytical and experimental studies confirm a relation between increased consumption of certain foods, essential to the MD, such as olive oil, nuts, fish, fruits and vegetables, fiber, and whole grains, and moderate consumption of alcohol and a reduced risk of major chronic degenerative diseases and other favorable health outcomes (Table 1). In the Prevención con Dieta Mediterránea (PREDIMED), a large intervention trial, large reductions in incident cardiovascular disease (CVD) and diabetes mellitus were observed in the MD groups as compared to the low-fat control group [9••, 10••, 11]. Systematic reviews have also investigated the effects of MD on cardiovascular risk factors in overweight, obese, and type 2 diabetic (T2D) subjects and have reported health improvements [12–14].

**Table 1:** General dietary recommendations based on Mediterranean diet principles

Daily intake		Weekly		Rarely (<2 times/week to several times per month)
Food item	Goal	Food item	Goal	Food item
Olive oil	≥4 tbsp/day	Nuts	3–7 serv/week	Red and processed meat
Yogurt	≤2 serv./day	Fish/seafood	≥3 serv/week	Commercial and refined sweets^c^
Fresh fruits	≥3 serv/day	Eggs	2–3 serv/week	Soda and sugary drinks^c^
Vegetables	≥3 serv/day	Poultry	2–3 serv/week	Refined carbohydrates^c^
Whole and refined grains^a^	Women 75 g/day, men 90 g/day	Legumes	≥3 serv/week	
Fresh herbs, allium family (onion, garlic)	≥twice/day	“Sofrito^b^”	≥2 serv/week	
		Wine with meals (optionally, only for habitual drinkers)	≥7 glasses/week	

Goals adapted from Wang et al. [54] and Estruch et al. [55]

^a^Whole grains brown rice, popcorn, and any grain food with a carbohydrate to fiber ratio no more than 10:1

^b^Sofrito is a sauce made with tomato and onion and/or garlic, slowly simmered with olive oil

^c^Discouraged

As with the general population, overweight, obesity, and other metabolic conditions are on the rise in the workplace and are recognized significant contributors to decreased productivity, work accidents, comorbidities, disability, and mortality [15]. Hence, interventions which prevent cardiometabolic conditions are clearly a leading priority for workplace health promotion. In this regard, recent work has shown the effectiveness of the Mediterranean diet in preventing CVD is roughly equivalent to that of lipid-lowering medications, but without adverse effects, while providing added benefits on weight control, diabetes prevention, and decreased cancer risk [16–18]. Table 2 summarizes the health benefits of MD and type of supporting evidence. Nonetheless, the MD, as a potential weapon against such diseases, is often overlooked and not adequately promoted in workplace settings.

**Table 2:** Summary of the health benefits of Mediterranean diet

Evidence supporting health benefit of Mediterranean diet	Magnitude of effect of Mediterranean diet on disease process	Type of study evidence
Reduction of CHD mortality	20–40 % ↓ in risk	Observational-longitudinal [1, 27], randomized controlled Trial [11, 30]
Reduction of CVD morbidity	25–45 % ↓ in risk	Observational-longitudinal [1, 32, 36], randomized controlled trial [30]
Reduction in the incidence of diabetes mellitus	25–30 % ↓ in risk	Observational-longitudinal [27], randomized controlled trial [11, 48]
Decrease in body weight, BMI, and abdominal circumference	Up to 40 % reduction	Cross-sectional [1, 45], observational-longitudinal [23, 41], randomized controlled trial [42, 48]
Improvement of metabolic syndrome components	Variable, 30–40 % reduction	Uncontrolled interventions [41], cross-sectional [45], observational-longitudinal [36], randomized controlled trial [46, 48]
Reduction of cancer mortality	20–30 % ↓ in risk	Observational-longitudinal [56, 57], randomized controlled trial [12]
Reduction of all-cause mortality	17–25 % ↓ in risk	Observational-longitudinal [56, 58, 59]

Recent efforts have attempted to improve workplace eating patterns, meal frequency, and the source of meals in order to promote healthier food habits. A recent cross-sectional study from Finland of over 4500 workers demonstrated that 30 % of employees ate at a worksite canteen daily, whereas 30 % of men and 45 % of women ate packed lunches [19]. Such nationally representative cross-sectional population surveys show that creating healthy catering services can potentially lead to more healthy food habits. Employees eating lunch at a worksite canteen offering healthy options tend to make food choices closer to nutritional recommendations as compared to those not using catering services to the same degree [19]. Similar data is available from US public sector employers as well. The North Carolina “HeartSmart” study and Arkansas “Healthy Employee Lifestyle Program” both found an increased consumption of fruit and vegetables among state employees after program participation [20, 21]. Such programs may provide easy, cost-effective solutions to improve employee health in both public and private sectors, thereby reducing the effect of obesity and unhealthy dietary habits on employee health and health care costs.

Meta-analyses of dietary behavior interventions at workplaces have shown both an increase in healthy eating (increased fruit and vegetable intakes by one-half serving/day) [22] and significant reductions of body weight (nine studies; mean difference [MD] −1.19 kg [95 % confidence interval (CI) −1.64 to −0.74]), body mass index (BMI) (11 studies; MD −0.34 kg m^−2^ [95 % CI −0.46 to −0.22]), and body fat (three studies; MD −1.12 % [95 % CI −1.86 to −0.38]) [23]. A 1-year intensive worksite program in a large hospital demonstrated that cardiovascular biomarkers such as mean cholesterol and blood pressure were improved at the end of follow-up [24]. Hence, worksite programs can successfully initiate weight loss and cardiovascular risk reduction among employees. The MD, which has been thoroughly studied and which offers major CVD protective benefits, however, has been implemented only infrequently at workplaces. This review will outline features of the MD, provide evidence about its protective role in several chronic diseases, and discuss its potential use in workplace health promotion strategies.

## Cardiometabolic Disease and the Mediterranean Diet

CVD remains the leading cause of death and disability in most developed countries, providing a strong incentive to improve dietary practices for CVD prevention. Several groups have demonstrated an association between MD adherence and a reduced prevalence of traditional CVD risk factors such as hypertension, dyslipidemia, and diabetes mellitus [25–27]. In a cross-sectional analysis of 3204 asymptomatic high-risk patients, an inverse relationship between MD score and the presence of CVD risk factors was observed [28]. Moreover, significant reductions in blood pressure, inflammatory markers, total cholesterol (TC), low-density lipoprotein, and triglyceride levels were observed in patients with mild dyslipidemia following a 4-month trial of MD [29].

The Lyon Diet Heart Study demonstrated the effect of a “Cretan Mediterranean” diet prioritizing monounsaturated fatty acid (MUFA) intake in reducing recurrence rates after a first myocardial infarction (MI) by more than 70 % [30]. Meta-analyses have shown that MD and increased nut consumption were also protective against the incidence of coronary heart disease (CHD) [31]. In the Nurses’ Health Study cohort, an association between a high MD score and a reduction in CHD and stroke incidence (relative risk 0.61 and 0.71, respectively) was demonstrated in a sample of 74,886 women aged 30–63 years [32]. Likewise, the international multicenter EPIC study confirmed that higher adherence to MD was associated with lower CHD risk in a Dutch cohort (*n* = 40,011), [33] a Spanish cohort (*n* = 41,078), [34] and in a Greek cohort (*n* = 23,601) and was also associated with significantly decreased stroke-related morbidity (hazard ratio 0.60) [35]. Comparative analysis of a case–control study in 1000 Greek participants demonstrated that adoption of the MD protected against acute coronary syndromes and stroke occurrence (hazard ratio 0.50) [36]. In another cross-sectional study involving 372 Greek patients with chronic heart failure, consumption of an MD improved systolic and diastolic ventricular function [37].

The associations of MD with improved cardiovascular health are not limited to Mediterranean nations. The Northern Manhattan Study, a cohort study involving 2568 participants, also found that higher adherence to a MD correlated with reduced risk of myocardial infarction, vascular disease, and stroke [38]. Recently, a prospective cohort involving over 17 thousand patients with CHD from the Health Professionals Follow-Up Study and the Nurses’ Health Study showed that a higher adherence to the MD was associated with a lower risk of CHD and total mortality [39]. Moreover, a Danish cohort followed for 11 years (*n* = 1849, 11 years of follow-up) reported that an increasing MD score was negatively correlated with morbidity and mortality from myocardial infarction and all-cause death [40]. Hence, the MD is associated with a lower incidence of CVD events and reduced mortality in both Mediterranean and non-Mediterranean populations (see Table 2).

As with the general population, CVD is also prevalent in the workforce; however, little is known about the effects of Mediterranean-style diet among young working groups in non-Mediterranean countries. In line with the above disease prevention strategies, specific studies focusing on MD in the workplace setting have been conducted. In a Chilean longitudinal study of 12 months, Leighton et al. evaluated the feasibility of a worksite MD intervention and its effects on metabolic syndrome in a middle-aged healthy male population [41]. Specifically, 171 workers of the Santiago division of a metal-mechanic company servicing the mining industry were invited to participate. Of these, 145 workers were eventually enrolled and their level of physical exercise was not modified. Together with an educational program focused on the metabolic syndrome, a MD intervention at the company canteen was performed. During the intervention period, the workers received a salad bar (previously not available) with four different mixed salads every day, two options for main courses, and fruits as dessert. To encourage salad consumption, an attractive olive oil-based salad dressing containing herbs and spices was always available. In addition to the daily MD options, a vegetarian dish and an option of beef with rice for employees not interested in adhering to MD was available. Complementing the dietary changes, the participants attended educational talks at least four times during the intervention. While the intervention had no effect on body weight, significant improvements were observed in values for the following metabolic syndrome components: waist circumference, HDL cholesterol, and blood pressure. The most striking effect was observed in blood pressure; both diastolic and systolic arterial blood pressure values declined during the entire intervention (13 and 15 mmHg, respectively). Waist circumference decreased consistently and significantly along the study, and HDL cholesterol increased particularly over the initial 8 months of observation. Taken together, there was a 35 % decrease in metabolic syndrome prevalence in the workers. Possible explanations for the changes observed include the following: an increased MUFA/saturated fatty acid ratio approximately threefold along the intervention (due to a reduction in red meat and processed foods and increased use of MUFA oils); a higher intake of vegetables and whole grains associated with reduced blood pressure; and an increase in fish consumption and possibly a reduction in overall caloric intake.

Data from workplace dietary interventions are often limited by study duration. Shai et al. conducted a 2-year workplace-based study in Israel, called the Dietary Intervention Randomized Controlled Trial (DIRECT) [42]. Over 300 moderately obese participants (mean BMI = 31) were randomly assigned to one of three weight-loss plans: a low-fat, restricted-calorie diet; a Mediterranean, restricted-calorie diet; or a low-carbohydrate diet without calorie restriction. The group provided nutrition labeling and diet-group color coding daily in the workplace cafeteria and also performed a spousal education program [43]. With 85 % adherence rates, after 2 years, the mean weight loss was 2.9 kg in the low-fat group, 4.4 kg in the MD group, and 4.7 kg in the low carbohydrate group. There was also a significant diet-induced decrease in carotid-vessel wall volume [44]. After the 2-year intervention was completed, the participants were followed for an additional 4 years. For the entire 6-year period, the total weight loss was 0.6 kg in the low-fat group, 3.1 kg in the Mediterranean group, and 1.7 kg in the low carbohydrate group (*p* = 0.01 for all comparisons). In the MD group, reductions in triglyceride levels from baseline (21.4 mg per deciliter [0.24 mmol/L], *p* = 0.03) and total cholesterol (13.9 mg per deciliter [0.36 mmol/L], *p* = 0.001) were also observed.

More recently, Yang et al. conducted a cross-sectional study in a cohort of 780 career male firefighters, investigating a modified Mediterranean diet score (mMDS) to assess MD adherence and its associations in a population of US Midwestern firefighters [45]. The cohort included young, occupationally active career male firefighters from 11 fire departments in two Midwestern states. MD diet adherence was assessed from responses to a life-style questionnaire, and CVD biomarkers were measured during the firefighters’ baseline medical evaluations. Participants in the highest quartile of mMDS compared to the lowest quartile had a 35 % lower risk for the presence of an additional metabolic syndrome component after adjustment for age and physical activity (odds ratio [OR] 0.65 after adjusting for age and physical activity, 95 % confidence interval [CI] 0.44–0.94, *p* = 0.039), suggesting that adherence to a Mediterranean-style diet in a young and active cohort could potentially reduce CHD-risk clustering and metabolic syndrome prevalence. Furthermore, for every unit increase of mMDS, a statistically significant decrease in mean low-density lipoprotein (LDL) cholesterol and increase in HDL cholesterol and a decrease in the triglyceride/HDL ratio were observed. Lastly, participants in the highest quartile of mMDS reported a significantly reduced odds of weight gain over the last 5 years (OR adjusted by age, BMI, and physical activity 0.57, 95 % CI 0.39–0.84, *p* = 0.01). These observed relationships support the potential effectiveness of a Mediterranean-style diet in younger, working cohorts and justify future intervention studies.

MD also has been shown to improve metabolic diseases such as insulin resistance, dyslipidemia, and the metabolic syndrome. In one of the first intervention studies of its kind, Esposito et al. studied the effects of a MD on patients with the metabolic syndrome, assessing endothelial function, vascular inflammatory markers, and persistence of metabolic syndrome components. After 2 years of intervention, only 44 % of the patients in the MD group still had the metabolic syndrome, while the patients in the control group did not show a significant reduction [46]. In 848 Greek patients with a first ischemic cardiac event and 1078 persons without evidence of cardiovascular disease, Pitsavos et al. studied subjects with metabolic syndrome and observed that the MD was associated with a 35 % reduction in coronary risk [47]. A previous meta-analysis of 50 studies (13 cross-sectional, 2 prospective, and 35 clinical trials; *n* = 534 906) found that adherence to the MD was associated with a reduction in the risk of metabolic syndrome (hazard ratio 0.50), as well as with improvements of systolic and diastolic blood pressure and reduction in triglycerides [36]. In another study from the same group, weight loss and improvements in glycemic control were significantly greater in overweight patients with newly diagnosed T2DM that were assigned to a low-carbohydrate, Mediterranean-style diet compared with those adhering to a low-fat diet [48]. The initiation of anti-diabetes medications drugs was also delayed in the MD-treated group as compared to controls [48]. Fish and omega-3 fatty acids, principal components of the MD, have been associated with a lower risk of cardiovascular disease [49]. Mediterranean diet improves blood pressure and lipid profile, decreases risk of thrombosis, improves endothelial function and insulin resistance, and reduces plasma homocysteine concentrations [50, 51]. Preliminary results of a Spanish intervention study (PREDIMED) showed after 3 months that two variants of MD supplemented either with olive oil or nuts yielded improved beneficial changes in most risk-factor outcomes measured [11]. Compared with a low-fat control diet, changes were found in plasma glucose levels, in blood pressure, and in HDL cholesterol. The conclusion was that MD supplemented either with nuts or olive oil is better than a low-fat diet to reduce metabolic syndrome. Atherogenic lipoproteins, small LDL, large very low-density lipoprotein (VLDL), and apolipoprotein B were also shown to be reduced in cohorts following a Mediterranean-style, low-glycemic-load diet [52, 53].

## Conclusions

The impact of specific dietary patterns and foods on the development of chronic diseases has been well-documented in a plethora of studies. Specifically, the MD has been shown to be associated with lower insulin resistance and endothelial inflammation, improvement in blood pressure and lipid profiles, and metabolic syndrome, which are likely to explain documented reductions on the risk of CVD morbidity and mortality and all-cause mortality. Furthermore, MD intake is associated with a decreased risk of various cancers. Recent efforts have attempted to modify workplace eating patterns, meal frequency, and the source of meals in order to promote healthier dietary habits at work, as well as to identify and overcome potential barriers. Limited workplace experience and evidence for population behavior change strategies support positive effects using modalities such as incentives, no-cost supplements of key foods (e.g., olive oil, nuts), pricing strategies, and targeted labeling and positioning of healthier foods. Table 3 summarizes some suggested dietary improvement goals and potential strategies. While limited workplace data is available, it supports benefits from workplace interventions, and further large clinical trials are needed to develop the most appropriate dietary strategies and interventions in the workplace. Given the wealth of evidence supporting the MD and its potential cardioprotective role, future emphasis on the role of MD in the workplace is likely to be a promising strategy to improve metabolic and cardiovascular health outcomes.

**Table 3:** Common poor dietary habits, goals and objectives for changing these patterns, and potential strategies for workplace dietary interventions

Challenges in the workplace [45]	Goals/objectives	Potential strategies
High intake of soda and sugary beverages	Increase water, zero-calorie beverage consumption	Group/individual education, color code drink choices, vending machine strategies
Lower quality oils used at work	Make olive oil the primary fat for cooking and condiments	Group/individual education, supplement workplace canteens with extra virgin olive oil
Low vegetable and legume intake	Increase average intake to three to four servings/day	Group/individual education, use olive oil to improve flavor, supplement workplaces with salad bars and legume flour pasta
Lower quality starches used at work	Increase intake of unrefined whole grains	Group/individual education, supplement workplace canteens with whole grain/legume flour pasta
Low consumption of fish	Increase average intake to ≥2 portions/week	Group/individual education, supplement workplace canteens with fish
High fast-food consumption	Reduce intake of unhealthy fast-food	Group/individual education, provide healthy options, color code food choices
Low nut intake	Increase to ≥1 serving of tree nuts/day	Group/individual education, supplement canteens with tree nuts

Strategies adapted from Artinian et al. [60] and Mozaffarian et al. [61]
